# Cancer cell reprogramming to identify the genes competent for generating liver cancer stem cells

**DOI:** 10.1186/s41232-017-0041-x

**Published:** 2017-05-18

**Authors:** Kenly Wuputra, Chang-Shen Lin, Ming-Ho Tsai, Chia-Chen Ku, Wen-Hsin Lin, Ya-Han Yang, Kung-Kai Kuo, Kazunari K. Yokoyama

**Affiliations:** 1 Graduate Institute of Medicine, Kaohsiung Medical University, Kaohsiung, 807 Taiwan; 2 Department of Biological Sciences, National Sun Yat-sen University, Kaohsiung, 805 Taiwan; 3 Center of Stem Cell Research, Kaohsiung Medical University, Kaohsiung, 807 Taiwan; 4 Department of Surgery, Department of Medicine, Kaohsiung Medical University, Kaohsiung, 807 Taiwan; 5 Center of Infectious Diseases and Cancer Research, Kaohsiung Medical University, Kaohsiung, 807 Taiwan; 6 Research Center for Environmental Medicine, Department of Medicine, Kaohsiung Medical University, Kaohsiung, 807 Taiwan; 7 Faculty of Molecular Preventive Medicine, Graduate School of Medicine, the University of Tokyo, Tokyo, 113-0033 Japan; 8 Faculty of Science and Engineering, Tokushima Bunri University, Sanuki, 763-2193 Japan

**Keywords:** c-JUN oncogene, Induced pluripotent stem cells, Liver cancer, OCT4, Reprogramming

## Abstract

The cancer stem cell (CSC) hypothesis postulates that cancer originates from the malignant transformation of stem/progenitor cells and is considered to apply to many cancers, including liver cancer. Identification that CSCs are responsible for drug resistance, metastasis, and secondary tumor appearance suggests that these populations are novel obligatory targets for the treatment of cancer. Here, we describe our new method for identifying potential CSC candidates. The reprogramming of cancer cells via induced pluripotent stem cell (iPSC) technology is a novel therapy for the treatment and for the study of CSC-related genes. This technology has advantages for studying the interactions between CSC-related genes and the cancer niche microenvironment. This technology may also provide a useful platform for studying the genes involved in the generation of CSCs before and after reprogramming, and for elucidating the mechanisms underlying cancer initiation and progression. The present review summarizes the current understanding of transcription factors involved in the generation of liver CSCs from liver cancer cell-derived iPSCs and how these contribute to oncogenesis, and discusses the modeling of liver cancer development.

## Background

The cancer stem cell (CSC) hypothesis initially proposed for leukemia by J. Dick is now accepted [1–3]. CSCs represent a small subset of cells within a tumor that are endowed with stem-like properties such as the ability for (i) self-renewal, (ii) pluripotency, (iii) tumor formation, and (iv) drug resistance [4, 5]. Importantly, CSCs are thought to be responsible for tumor initiation, recurrence, and metastasis through the reduced sensitivity of cancer cells to chemotherapy compared with that of the original tumor cells [6]. Growing evidence about liver CSCs confirmed their resistance to therapeutic drugs such as cisplatin, 5-fuluorouracil, and so on [7].

The primary strategy for inducing liver CSCs is to first enrich the cells using classical stem cell markers of stem cells such as CD13, CD24, CD44, CD47, CD90, CD133, epithelial cell adhesion molecule, and OV6, and then to apply functional methodologies such as side-population analysis, the ALDEFLUOR^TM^ assay, the sphere formation, and so on [8–11]. This cell population is then transplanted into immuno-deficient mice to examine its in vivo tumorigenic potential [7–9], and the cells are studied further according to their expression of various genes or signals such as Wnt, Notch, Hedgehog, Transforming growth factor β, epithelial mesenchymal transition (EMT)/mesenchymal epithelial transition (MET) signaling, epigenetic regulators, and microRNAs. The putative CSC subpopulation capable to initiating tumor development at lower cell numbers are tested further for self-renewal capacity using serial dilution of cells to identify the CSCs. Cancers are also generated as a consequence of transformation of the “driver” mutation at initiation [12–15], and then positive selection and clonal progression lead to the accumulation of “passenger” mutations required for additional growth advantages. It is generally accepted that a novel strategy is needed for the functional evaluation of both putative driver and passenger mutations, and for studying their molecular dynamics underlying cancer development.

Current cancer cell-reprogramming techniques such as somatic cell nuclear transfer [16] and the generation of induced pluripotent stem cells (iPSCs) [17–19] are used to identify oncogenic genes. In 2006, Yamanaka et al. [17] reported on the reprogramming of somatic cell into induced pluripotent stem cell-like cells (iPSLCs). The success in reprogramming a somatic cell into a stem cell-like state has led to the idea of reprogramming malignant cells back to their original state, which is well before the oncogenic transformation occurs. The generation of iPSCs from cancer cells may provide tools for exploring the mechanisms of tumor initiation and progression in vitro, for studying the heterogeneity and origin of CSCs, and for producing cancer type-specific drug discovery. However, these reprogramming methods remain a challenge because of the cancer-specific epigenetic state and chromosomal aberrations of cancer cells.

The epigenetic memory of the original cell type is important to reprogramming and is closely related to the inefficient reprogramming caused by failure of reset the epigenome to an embryonic stem cell (ESC)-like state [20]. The epigenetic state is reversible, but attempts to reprogram cancer cells have produced incomplete resetting of the cancer-associated epigenome because of tumor heterogeneity and further accumulation of oncogenic mutations. Therefore, cancer cell reprogramming is currently limited to certain cancer types and cancer-specific marks in the epigenome, which impede successful reprogramming, and the underlying mechanism has not been fully elucidated. The technique of cancer cell reprograming is one possible approach for identifying the committed genes of CSCs and for studying the mechanisms underlying the transcriptional, translational, and epigenetic inheritance of cancer development. The characterization of epigenome and tumor suppressor gene complexes, such as p53, p21^Cip1^, p27^Kip1^, the Ink4 family, and the polycomb repressive complex (PRC) might identify the possible candidates for genes for reprogramming of cancer cells to CSCs.

### Obstacles to cancer cell reprogramming

The reprogramming of cancer cells is less successful than the reprogramming of somatic cells [21–23]. Despite the presence of genetic alterations, melanoma cells can be reversed to the pluripotent state, such as that of the ESC, by nuclear transfer [24] (Table 1). However, the cells involved in other malignant cancer types, including breast cancer, leukemia, and lymphoma, cannot be reprogrammed, which suggests the presence of unknown barriers to reprogramming [23, 25, 26]. Similarly, embryonic carcinoma (EC) cell lines can be reprogrammed by nuclear transfer and exhibit normal preimplantation development. However, abnormal phenotypes of the clones occur, which suggests that EC cell lines possess a unique set of modifications in the epigenetic state that cannot be reprogrammed by altering the developmental potential [27]. The limited number of studies that have reported successful derivation of iPSCs from cancer cells supports the notion that the existence of cancer-specific genetic and epigenetic states hinders successful reprogramming independently of the complexity of the techniques used for reprogramming. Reprograming studies using patient-derived cancer cells have produced solid evidence of the capacity for reversal of the malignant phenotype, and this method holds great promise for breaking the seemingly irreversible state associated with cancer.

**Table 1:** Summary of studies of reprogramming of cancer cells from induce pluripotent stem cell technology. We have modified the summary of the table reported by Camare et al. [25, 26]

CSCs examples	Original cells	Methods of reprogramming	Karyotype	Chimeras	Teratoma/tumor formation	Drug sensitivity
Utikal et al. (2009) [65]	Mice melanoma R454 (rasinduce cells)	Lentivirus OKM	Trisomy chromosomes 8 and 11	Yes	Yes	No tumor in the absence of Dox
Carette et al. (2010) [42]	Human leukemia KBM7 (CML)	Retrovirus OSKM	Tetraployd, chromosomes 9 and 22 Ph(+)	Not applied	Yes	Cell type specific drug sensitivity
		Retrovirus OSK (incomplete reprogramming)				
Miyoshi et al. (2010) [43]	Human gastrointestinal cancer cells	Retrovirus and lentivirus + lipofectamine OSKM	Abnormal	Not applied	Yes	Post iPSC cells—more sensitive to 5-Fu and differentiation inducing drug
Kuo et al. (2016) [59]	Human HepG2 liver cancer cells and mouse hepatocytes-iPSCs	Lentivirus OSKM + shp53RNA (lentivirus O + c-JUN for direct reprogramming)	Abnormal	Not applied	Yes	Yes

### Stemness characteristics and oncogenic functions of CSCs

Generation of iPSCs from cancer cells requires the identification of genes that regulate stem cell self-renewal and pluripotency such as *OCT4, SOX2,* and *NANOG*. CSCs require oncogenes or tumor suppressor genes to express oncogenic functions. Both expression states of stemness genes and oncogenes/tumor suppressor genes should be reorganized at the chromatin state by their regulators of epigenetic modification. Thus, three classes of gene sets might provide clues for understanding the reprogramming of cancer cells.

OCT4 is known to be overexpressed in hematological cancers, seminomas, and cancers of the bladder, brain, lung, ovary, pancreas, prostate, kidney, and testicle [28]. OCT4 works synergistically with SOX2 via direct binding to transactivate the target genes [29]. Both *SOX2* and *OCT4* are activators of genes involved in pluripotency, including themselves and *NANOG* [30], and repressors of genes involved in differentiation [31, 32]. Both SOX2 and OCT4 regulate their own transcription by binding the composite elements of SOX–OCT in their enhancers [33].

Overexpression of SOX2 is detected in recurrent prostate cancer, head and neck squamous cell carcinoma, glioblastoma, small-cell lung cancer, and cancers of the breast, liver, pancreas, and stomach [33]. Overexpression of SOX2 increases cell proliferation via cyclin D3, and represses cell cycle regulators such as p21^Cip1^ and p27^Kip1^ [34]. SOX2 promotes the invasion, migration, and metastasis of melanoma, colorectal cancer, glioma, and cancers of the stomach, ovary, and liver through the activation of matric metalloproteinases family, and phosphatidylinositol 3-kinase (PI3K)–RAC-α serine/threonine kinases (AKT)–mammalian target of the rapamycin signaling pathway [35–37].

NANOG is overexpressed in oral squamous cell carcinoma and other types of cancers [38]. NONOG is capable of maintaining pluripotency of ESCs independently of the leukemia inhibitory factor-signal transducers and activator of transcription pathway, which is different from the case of OCT4 [38, 39]. NANOG also controls the cell cycle and proliferation by directly binding to the cyclin D1 promoter for transactivation [40]. NANOG induced the expression of cancer-related genes like CD133 and aldehyde dehydrogenase 1A1 [41]. These stemness transcription factors of SOX2, OCT4, and NANOG co-occupy the promoter regions of about 350 genes in the genome, and OCT4 occupies more than 90% of the promoter regions bound by the OCT4 and SOX2 in human ESCs. These findings suggest that the OCT4–SOX2–NANOG axis is the key cascade for stemness [31].

### Reprograming of cancer cells using iPS technology

It has been proposed that oncogenes and tumor suppressor genes should be activated or repressed to generate CSCs. However, the actual oncogenes that can generate CSCs have not been characterized.

Carette et al. [42] reprogrammed a cell line derived from chronic myeloid leukemia (CML) by infecting them with a retrovirus that induced the expression of OCT4, SOX2, KLF4, and MYC (OSKM) followed by the subcutaneous injection of the CML-iPSCs into nonobese/diabetic severe combined immunodeficient (NOD-SCID) mice [Table 1]. They found that the teratomas produced contained differentiated cells in three germ layers, which indicated pluripotency. Whereas the parental CML cell lines were dependent on the BCR–ABL pathway, by contrast, the CML iPSCs were independent of this BCR–ABL signaling and showed resistance to imatinib. However, Cratte et al. did not identify the signaling pathway involved in the suppression of this BCR–ABL cascade. Miyoshi et al. [43] reported on the reprogramming of gastrointestinal cancer cell lines into iPSCs through the OSKM method [Table 1]. Tumors were generated by parenteral injection of gastrointestinal cancer cells into NOD-SCID mice, but not by injection of differentiated cells arising from the iPSCs. These iPSCs expressed increased levels of tumor suppressor genes such as p16^Ink4a^ and p53 upon differentiation.

Striker et al. [44] reported the reprogramming of glioblastoma (GBM) cells to neural stem cells (NSCs) by PiggyBac transposon vectors that expressed OCT4 and KLF4. In these GBM iPSCs, the widespread resetting of epigenetic methylation occurred in cancer-specific methylation variable positions, the GBM tumor suppressor gene CDKN1C (p57^Kip2^), and testin LIM domain protein (TES). The neural progenitor cells (NPCs) differentiated from GBM iPSCs resembled aggressive GBM cells when transplanted into the adult mouse brain [44]. By contrast, non-neural mesodermal progenitors from GBM iPSCs with sustained expression of TES and CDKN1C formed benign tumors, and failed to infiltrate the surrounding regions. These findings suggest that DNA methylation is critical to the expression of these particular genes. Kim et al. [45] generated the iPSC-like cells from pancreatic ductal adenocarcinoma (PDACs) by introducing the genes encoded *OSKM*. One cancer iPS-like clone harbored classical PDAC mutations, including kRAS and p16^Ink4a^ heterozygous deletions and decreased SMAD copy number, and retained the chromosomal alterations seen in the parental cells, and differentiated into all three germ layers during in vitro differentiation in the descendants, although the neural lineages were underrepresented. An in vivo teratoma showed that the iPSC-like cells generated multiple germ layers tissues but preferred to generate endodermal ductal structures.

DNA methylation is another critical epigenetic change. However, the reprogramming of cancer cells into iPSCs shows that only about 50% of cancer-associated epigenetic defects, as defined by a comparison between normal and tumor cells, are stably reset, which suggests that locus-specific changes in the epigenome are required. Reconfiguration of the network of cell-fate transcription factors and downstream developmental epigenetic mechanisms, may effectively silence the cancer-promoting pathways essential for uncontrolled proliferation and infiltration. However, the reciprocal interaction of epigenetic changes and driver mutations of cancer need to be explored in greater detail.

Funato et al. [46] used the pluripotent stem cells to study the role of driver mutations in modeling pediatric brain tumors. Differentiation of human ESCs into NPCs and induced cellular transformation through the overexpression of a constitutively active PDGFRA, TP53 knockdown, or expressing the K27M mutant of histone H3.3 found in pediatric gliomas. Moreover, induced NPC transformation was induced by the combination of different mutations typically observed in human gliomas that collectively affect the signaling of the PI3K, MAPK, and p53 pathways [47].

BMI1 of PRC1 has been shown to be involved in the maintenance and/or self-renewal of many types of stem cells, including embryonic, neural, hematopoietic, and prostate stem cells [48]. BMI1 promotes the proliferation of leukemic stem cells in a mouse model [49], and activates the self-renewal ability of NSCs [50]. BMI1 is known to be directly responsible for the regulation of multiple targets such p16^Ink4a^ and p14^Arf^ [51] and to bind directly to the promoter of *PTEN* gene, which results in the activation of the PI3K–AKT signaling and subsequent stabilization of SNAIL to induce the EMT [52]. BMI1 also occupies the cadherin promoter, which causes E-cadherin repression [52] and cooperates with TWIST1 to promote cancer dedifferentiation and metastasis [53]. In endometrial cancer cells, the loss of BMI1 results in the reduced expression of SOX2 and KLF4 [54]. Overexpression of BMI1 correlates with overexpression of NANOG, high tumor grade status, and increased self-renewal in breast adenocarcinomas [55].

Recently, Kaufhold et al. [56] reported the association of Yin Yang 1 (YY1) and CSC transcription factors and that YY1 might be a transcriptional repressor that acts on CSC-associated transcription factors such as *BMI1, SOX2*, and *OCT4*. They also proposed the existence a regulatory loop involving crosstalk between the nuclear factor kB–PI3K–AKT pathway and the downstream controls of target gene products such as YY1, OCT4, SOX2 and BMI1. Taken together, these findings suggest that most of the genes critical for cancer reprogramming belong to the families of (i) stemness genes, (ii) oncogenes/tumor suppressor genes, and (iii) epigenetic-related genes of DNA or histone modification, and (iv) the INK4 locus mediated by PRC family.

**Fig. 1:**
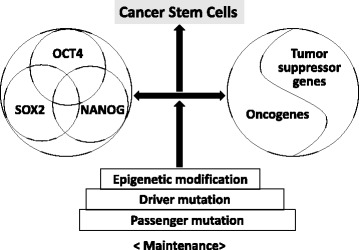
Schematic representation of hypothetical two-hit theory for crosstalk to generate cancer stem cells by reprogramming. Hypothetical two-hit theory for induction of cancer stem cells was represented. Stemness factors (OCT4, SOX2, and NANOG) and oncogene/tumor suppressor genes (oncogenes such as Myc, KLF4, c-JUN, kRAS, etc.; antioncogenes such as p53, Rb, PTEN, BMI1, EZH2, INK4 family, etc.) and epigenetic modification of DNA methylation and histone modification are required for generation of cancer stem cells by reprogramming. We have reported the feedback control of c-JUN and OCT4 is critical for generation of cancer stem like cells [59]. The oncogene c-JUN transactivated genes encoding OCT4, SOX2, and NANOG [60], and the genes of OCT4, SOX2, and NANOG formed the molecular circuitry for stemness and pluripotency [64], and then OCT4 upregulated the expression of c-JUN gene to form the feedback circuit [59]. Taken together, we hypothesize that these feedback circuit might be regulated by the family of the stemness genes and the family of cancer-related oncogenes or tumor suppressor genes

Thus, we hypothesized a “two-hit” theory for the generation of CSCs. The first hit introduces stemness and the second hit induces oncogenic features or repression of tumor suppressor function. Both are required for induction and maintenance of CSCs by epigenetic alterations induced by both genes. In this model, the reprogramming can be used as a platform for identification of the functional driver or passenger mutations, and modifications under the activation of stemness genes and the activation of oncogenes (or the repression of tumor suppressor genes) (Fig. 1).

### Selective plasticity of CSCs

Cell plasticity is a key issue for the reprogramming of CSCs. Mu et al. [57] and Ku et al. [58] independently reported the role of cell plasticity in cell identity, which allows cancers to thrive. It has been known that hormone deprivation therapy that suppresses androgen receptor (AR) signaling is one of the treatments for metastatic prostate cancer. However, prostate cancers can become resistant to these drugs by losing hormone dependence on androgen. Because androgen stimulates the growth of prostate cancer cells, decreased production of androgen and/or inhibition of the hormone’s action on prostate cancer cells can make a tumor shrink or grow more slowly. Mu et al. [57] reported that the induction of SOX2 expression subsequent to loss of RB1 and TP53 contributes to neuroendocrine differentiation and androgen independence of prostate cancer cells. Ku et al. [58] examined the effects of RB1 and TP53 deletion in a mouse model of metastatic prostate cancer (PTEN loss) and found that EZH2 inhibition restored enzalutamide responsiveness in RB1- and TP53-depleted and AR expressing LNCaP cells. The cell lineage plasticity was determined by the E2F-regulating genes such as SOX2 and EZH2. These data suggest that both tumor suppressor genes, such as RB1, TP53, and PTEN, and the cell cycle regulator E2F-controlled genes, such as SOX2 and EZH2, might be critical for determination of cell lineage plasticity.

### Clone selection of CSCs from iPSC-like cells

In an attempt to isolate the CSCs, we have used a new strategy to isolate the clone responsible for generating CSCs among the heterogeneous clones of iPSC-like cells derived from human hepatocyte cell lines [59]. Using the original four Yamanaka’s factors plus small hairpin TP53 plasmid, we have generated iPSC-like clones. To identify the possible CSC clones, we have reduced the number of cells that remain competent after tumor formation, as determined by the transplantation of colonies of xenograft-derived iPSC-like cells. One colony comprising about 200 cells finally generated tumors in more than 40% of transplants. We then characterized this colony to determine its tumor-forming capacity. This clone exhibited greater tumor-forming activity and other cancer-related activities such as sphere formation, colony formation, invasion activity, and drug resistance compared with the original liver cancer cells.

To gain further insight into the acquired CSC characteristics of this CSC colony, RNA sequencing was performed and showed that OCT4 and c-JUN expression was greater in this clone than in the original cancer cells. This experiment suggests that both OCT4 and c-JUN are key factors required for the feedback control of each other. Moreover, the two genes—one a stemness gene and the other an oncogene—were also competent in inducing CSCs derived from iPSCs from mouse hepatocytes infected with lentivirus that encoded OCT4 and c-JUN. This combination is interesting because *c-JUN* is an oncogene and *OCT4* is a stemness gene. By themselves, OCT4 and c-JUN showed less transformation activity, but the feedback between OCT4 and c-JUN increased the likelihood of cancer induction. Therefore, we hypothesize that both genes are required to generate liver cancer and that the feedback regulation of OCT4 and c-JUN might be critical for triggering CSCs.

### Future perspectives

This strategy of cancer reprograming is one possible approach for generating CSCs. Chang et al. [60] reported that c-JUN is activated in pluripotent stemness gene promoters such as OCT4, SOX2, and NANOG and promotes the EMT in head and neck cancer cells. We have introduced OCT4 and c-JUN into mouse iPSCs from normal hepatocytes to generate tumor formation [59]. These approaches to induce the feedback regulation of the stemness gene family and oncogene family might provide a novel approach to generate CSC-like clones. Elevated expression of OCT4 and c-JUN was observed in specimens taken from patients with liver cancers. These findings suggest that both genes are possible candidates as future therapeutic targets.

## Conclusions

In this review, we have described the methods for generating CSC-like cells from iPSCs from liver cancer cells. The critical point is to isolate the clone with strongest tumor-inducing activity. We have identified the genes of stemness and oncogenes as possible CSC target genes. This approach can now be extended to isolate CSCs and to generate disease- or cancer-specific models with distinct features. However, the efficiency of cell reprogramming from iPSCs to CSCs is lower and each CSCs has been shown to be heterogeneous. Moreover, oncogene induced plasticity including the CSC markers, and the microenvironments controlling this process have still not been elucidated, especially in the cases of the solid tumors [23, 61–63]. Further work is needed to identify and characterize CSC-like cells in other cancers and diseases, which will help to detect the driver and passenger mutations for generating cancers and genetic diseases. The study of the reprogramming of cancer cells should be encouraged to further progress the understanding of cancer and disease biology.

## Abbreviations

CSC: Cancer stem cell
EC: Embryonic carcinoma
EMT: Epithelial mesenchymal transition
ESC: Embryonic stem cell
GMB: Glioblastoma
iPSC: Induced pluripotent stem cell
MET: Mesenchymal epithelial transition
MMP: Matrix metalloproteinase
NOD SCID: Nonobese diabetic/severe combined immunodeficient
OSKM: OCT4, SOX2, KLF4 and MYC
PRC: Polycomb repressive complex
YY1: Yin Yang 1
